# Association of Addictive Substance Use with Polyvictimization and Acceptance of Violence in Adolescent Couples

**DOI:** 10.3390/ijerph18158107

**Published:** 2021-07-30

**Authors:** Isabel Cuadrado Gordillo, Guadalupe Martín-Mora Parra, Inmaculada Fernández Antelo

**Affiliations:** Department of Psychology and Anthropology, University of Extremadura, 06071 Badajoz, Spain; guadammp@gmail.com (G.M.-M.P.); iferant@unex.es (I.F.A.)

**Keywords:** dating violence, adolescents, polyvictims, alcohol use, marijuana use, acceptance of violence

## Abstract

Theoretical framework: The objectives of this study were to analyse the possible influence that some variables such as substance use (alcohol and marijuana) might have on relevant aspects related to violence in adolescent dating (victimization, frequency of violence and acceptance of violence). Methods: The sample included 2577 adolescents between the ages of 14 and 18. The instruments used were two questionnaires. The first identified and analysed the types and frequency of violence experienced by the victims, and their acceptance of violence. The second analysed the use of alcohol and marijuana in adolescents. Results: The results indicate that victims frequently take on the role of polyvictims, suffering aggression in up to more than five different forms at the same time. Furthermore, it was found that this phenomenon is precipitated by substance use, the frequency of abuse and the acceptance of violence in a cycle of mutual interaction.

## 1. Introduction

Violence in adolescent dating is a social health problem characterized by the perpetration of abusive behaviour (psychological, physical, sexual, and emotional) in adolescent couples [1]. It involves more frequent violence than the gender violence occurring in adult couples, although it may be less noticeable since it tends to manifest itself more often as psychological violence, blackmail and control on the part of the aggressors [2].

The reasons why this is the case have not been easy to establish, with it being of vital importance to consider several variables that consider the complexity of the phenomenon from different perspectives. In this sense, the relationship between violence and such cognitive and behavioural aspects as ambivalent sexism [3,4], or the mechanisms of moral disengagement [5,6,7,8], seems to be well established in different contexts. However, as well as the aforementioned variables, there are other key behavioural aspects specifically related to some of the habits that adolescents begin to present during this stage.

Adolescence is when the consumption of alcoholic beverages begins for a large percentage of individuals. More than 25% of adolescents of ages from 15 to 19 years consume alcohol, meaning a total globally of 155 million young people [9]. Likewise, from the age of 16 it is common for the joint use of several substances to be more frequent than the use of a single one [10]. Thus, the consumption of tobacco occurs in conjunction with alcohol in a significant percentage of adolescents, which increases the risk of involvement in behavioural and health problems [11].

The relationship between substance use and the development of violent behaviour is a fact that has been extensively researched. For instance, it has been noted that alcohol influences behaviour, since it establishes a tendency to overreact while reducing cognitive inhibitions, with a result being increased likelihood of violence [12,13] due to decreased control [14]. In the same line, it has been found that alcohol consumption increases both the severity of the aggressions [15] and the likelihood that these aggressions will cause the victim injury [16,17,18,19,20,21,22].

Regarding adolescent couples, substance use has also been linked to violent behaviour, both as a precedent and as a consequence. Some research has revealed that, among adolescents visiting the doctor who have a history of alcohol and tobacco use, approximately 55% reported violence in their dating relationship, whether as aggressor or victim [23]. Other studies have indicated that both the excessive consumption of alcohol and daily alcohol intake are linked to aggression [24] and victimization in interpersonal relationships [25], as well as in adolescent dating relationships [26]. It is also usual to find temporal proximity between the intake of alcohol and the perpetration of both physical and psychological violent behaviour among undergraduates [27]. It has even been noted that people presenting a dangerous level of alcohol consumption are at greater risk of involvement in dating violence, especially when this is preceded or followed by alcohol consumption [28].

Behaviour of both the aggressor and the victim may be altered by substance use. In the case of the aggressors, it has been found that alcohol is strongly related to the perpetration of psychological, physical, and sexual violence among undergraduates [29]. Subsequently, these results were confirmed [30] with the additional indication that excessive alcohol consumption is the only type of consumption that appears to be linked to the perpetration of the three main types of violence (physical, psychological, and sexual) among undergraduate dating couples. Other authors point to alcohol intake consistently being predictive of dating violence [31], and it has been noted that the perpetration of violence during dating is more likely to occur when alcohol is consumed in the form of “binge drinking” [32], with this fact being common to both men and women [31]. It has even been noted that people presenting dangerous levels of alcohol consumption have greater risk of involvement in dating violence, especially when this is preceded or followed by alcohol intake [33].

In the case of the victims, some authors have indicated the effect that alcohol has both cognitively and physically; it lowers the perception of risk which increases the danger of victimization [34]. Testa & Livingston complemented these results in noting that the victim’s consumption of alcohol also diminishes their ability to escape or resist [35]. An earlier study [36] confirmed that binge-drinking alcoholic beverages during adolescence increases the chances of becoming a victim of bullying or of dating violence.

Since violence in adolescent dating is a complicated phenomenon, simple explanations based on a single variable seem to be insufficient. In this sense, it is more appropriate to consider complex explanations that take into account not only the individual influence of certain aspects, but also the interactions established between different variables. In this observed complexity, two other key factors are the victim’s acceptance of violence and the frequency with which such violence occurs. Indeed, it is common to find that adolescent girls have an altered perception of abusive behaviour, which largely normalizes violence [37].

It can therefore be said that the victims integrate many variables and characteristics in some way related to victimization. Nonetheless, the way in which these variables combine to ultimately influence the victim’s role has, paradoxically, not been so widely studied. In this context, and from a complex perspective, the present study aims to construct an analysis of victimization that integrates the possible association of certain variables, such as the use of substances (alcohol and marijuana) that are relatively common among some adolescents, with other relevant aspects such as victimization, the frequency of abuse, and the victims’ acceptance of violence. It is hoped with this to enlarge the theoretical framework explaining this phenomenon, as well as to contribute to the considerations necessary to establish effective prevention and intervention programs. With this objective, the following hypotheses are proposed:

**Hypothesis** **1** **(H1).** *The consumption of alcohol and marijuana affects victimization in adolescent dating violence*.

**Hypothesis** **2** **(H2).** *The frequency and acceptance of violence are related to victimization in adolescent dating violence*.

**Hypothesis** **3** **(H3).** *The consumption of alcohol and marijuana, and the frequency and acceptance of violence, predict victimization in adolescent dating violence*.

## 2. Material and Methods

### 2.1. Participants

The sample of this cross-sectional research study consisted of a total of 2577 adolescents (44.8% boys), aged between 14 and 18 years. The participants were selected randomly and proportionally through the development of a process stratified into different stages. The said process focused on the random selection of a group of pupils of Lower Secondary Education (ESO) and Upper Secondary Education (Baccalaureate and students from training cycles) from different geographical areas of the Region of Extremadura (the provinces of Cáceres and Badajoz) in Spain. These areas cover the north, south, east, and west of the Region, and include both rural and urban zones. This implies, as well, that the participants’ families present different cultural and socioeconomic characteristics, with the purchasing power of approximately half being medium-high, and of the other half medium-low. Similarly, about half of the participants’ families had higher education, while the other half had basic education. In each school, one academic year was selected from 3rd and 4th of ESO and 1st and 2nd of Baccalaureate. All the classes of this selected academic year participated in the study, with an average of 25 students per class. The ages of the participants varied depending on the academic year (3rd ESO: 14–15; 4th ESO: 15–16; 1st Baccalaureate: 16–17; 2nd Baccalaureate: 17–18).

### 2.2. Instruments

Two questionnaires were applied to carry out the study:

The CUVINO couple violence questionnaire [38]. This questionnaire comprises a total of 61 items. In their responses, the adolescents indicate the frequency with which, in their dating relationships, they have experienced eight different types of violence: detachment, humiliation, sexual, coercion, physical, gender, emotional punishment, and instrumental punishment. The participants have to respond on a Likert scale with five anchors: “never”, “sometimes”, “frequently”, “normally”, and “almost always”. The reliability analysis obtained with the present sample yielded values that varied from 0.66 to 0.83. These same items allow one to obtain the level of annoyance that violence within the couple causes them (or would cause in the case of never having suffered violence in a dating relationship). The acceptance or tolerance of dating violence that the adolescents in the study manifest is obtained from this index of annoyance, understanding that a high level of annoyance is identified with low acceptance, while a low level of annoyance corresponds to high acceptance. To this end, the questionnaire includes a Likert scale with five anchors ranging from “not at all” to “a lot”. The participants’ responses regarding annoyance can be grouped into eight factors that match the eight types of violence analysed. The reliability of this scale varied between 0.71 and 0.84.

The Habits and Lifestyle Questionnaire for Adolescents. This questionnaire comprises a total of 13 items. In their responses, the adolescents have to indicate such aspects as whom they usually hang out with, the activities they do in their free time, whether they drink in the street, and their habits regarding the consumption of alcohol and marijuana; in this last case responding on a Likert-type scale with the frequency of the habit. The scale has five anchors ranging from “No, I have never tried it” to “Every day”.

### 2.3. Procedure

In the first place, and before starting with the data collection, the Bioethics and Biosafety Committee of the University of Extremadura (Spain) approved the objectives set out in this research as well as the procedure, instruments and techniques to be used (Ref. 18/2017). Once this approval had been obtained, the data collection process began and was carried out throughout the 2018/2019 academic year. To this end, the researchers who formed part of the study went to each of the selected schools to give the adolescents the questionnaires in a pencil-and-paper format. Before entering the classrooms, the pertinent permits were requested from both the Regional Educational Administration and the different administration teams of each of the selected schools. Because the study involved minors, parental permission was requested in writing. To do this, a letter was sent to the parents describing the nature of the study and its objectives, as well as guaranteeing the anonymity and confidentiality of the responses. This letter was accompanied by an authorization form for the parents to sign allowing the participation of their children in the study. No financial reward was provided to either the schools or the participants.

When the researchers went to the different schools, they took with them the different questionnaires to administer. Once in the classroom, the researchers gave instructions to the adolescents for them to be able to complete the questionnaires, stressing that they were to be filled out individually and sincerely, as well as guaranteeing the adolescents’ anonymity. It was also made clear that participation in the research study was not compulsory, so that the adolescents could decide whether they wanted to complete the questionnaires or not. Two researchers remained in the classroom with the pupils to resolve any possible doubts that might arise, as well as to collect the questionnaires once the pupils had finished completing them. The participants had approximately 50 min to complete the questionnaires, which were then collected and kept until the later data analysis in separate boxes according to the educational level and the school they corresponded to.

### 2.4. Data Analysis

The analyses were carried out in three phases. In the first phase, descriptive analyses were carried out to identify the adolescents who were victimized in the different forms of abuse, additionally obtaining the frequency with which they had experienced the abuse. Next, having verified that many victims suffered from more than one aggression, the polyvictimization phenomenon was studied, considering that polyvictims were those adolescents who had suffered from more than one kind of abuse at the same time in their relationship. In the analysis, they were classified according to whether they had suffered two, three, four, or five or more abuses at the same time. In the second phase, a correlation study was carried out to reveal any relationships between the different variables considered in the study. This analysis helped us to understand the relationships established among the variables and guided the next phase of analysis.

In the last phase, an ANOVA was performed to check if there were differences between the different groups of polyvictims (polyvictims who consume alcohol, polyvictims who consume marijuana, and polyvictims who accept violence). Finally, the predictive capacity of the variables was analysed through a three-step hierarchical regression model that allowed a predictive model of polyvictimization to be constructed through the successive inclusion of the variables included in the study in three phases (frequency of violence and alcohol consumption, marijuana consumption, and acceptance of violence).

## 3. Results

First, those individuals who declared themselves to have suffered violence in an adolescent dating relationship were selected from the total study sample (non-victims = 1132; victims = 1430). The results show that, as the frequency of abuse increased, the number of victims decreased. However, it was also found that the victims did not experience one single type of abuse, but several types of violence at the same time, thus becoming polyvictims. The abuse types most noted by the victims were detachment (60.6%), coercion (37.2%), and emotional punishment (47.3%) (Table 1).

Second, the analysis of substance use indicated that the victims claimed to consume alcohol to a greater extent (males 85.4%; females 88.9%) than the nonvictimized adolescents (males 66%; females 67.3%). Similarly, victims reported smoking marijuana to a greater extent (males 29%; females 29.8%) than nonvictims (males 16.2%; females 13.2%) (Table 2).

Third, considering that the victims reported having suffered more than one type of abuse, a frequency analysis was carried out considering the number of abuses that these victims had experienced at the same time (two, three, four, or five or more types of abuse). This analysis also made it possible to observe the classification of these polyvictims depending on whether the abuses were committed “Sometimes” (victims who report having suffered violence “sometimes”), or “Frequently” (victims who report having suffered violence “normally” and “almost always” (Table 3).

As can be seen, in the “Sometimes” category of victims, the results are very close between the groups, showing that similar numbers of victimized adolescents reported experiencing two abuses at the same time (27.1%), five or more (25.3%), three (24.9%), and four (22.6%). In contrast, the polyvictims who were abused “Frequently” were mostly in the five or more types of abuse group (78.3%), showing that subjects who suffered violence most often were also those who report abuses in more categories at the same time, and were thus the most frequent polyvictims.

The results of the correlation analysis performed with those participants who reported having been victimized in one or more kinds of aggression (victims and polyvictims) allowed the relationships between pairs of scores to be studied, taking into account “victimization”, alcohol consumption, marijuana consumption, and “acceptance” of violence (Table 4).

The consumption of alcohol was found to correlate with “victimization” (0.36, *p* < 0.001), so that as victimization grew so did the consumption of alcohol. The consumption of marijuana was also found to correlate with “victimization” (0.29, *p* < 0.01), again so that as victimization grew so did the consumption of marijuana. The consumption of alcohol and marijuana correlated with each other (0.22, *p* < 0.05), with the consumption of marijuana being greater when the consumption of alcohol increased. Finally, it can be observed that the “acceptance” of violence increased as victimization, alcohol use (0.20, *p* < 0.05), and marijuana use (0.18, *p* < 0.05) increased.

Once the relationships between the variables included in the study had been checked, an analysis of variance (ANOVA) showed that the mean scores of the polyvictims in their consumption of alcohol and of marijuana differed depending on the number of aggressions they had suffered during that same time. This analysis also indicated how the “acceptance” of violence differed depending on whether victims had suffered abuse in one, two, three, four, or five or more categories (Table 5).

Specifically, the post hoc tests indicate that differences between the polyvictims were found in those who suffered two modalities of abuse compared to those who suffered three in the cases of alcohol use (−0.19, *p* < 0.05) and acceptance of violence (−0.21, *p* < 0.05). The differences between the group of polyvictims who suffered four types of abuse and those who experience three types were found in the cases of the acceptance of violence (−0.26, *p* < 0.05) and of alcohol (−0.20, *p* < 0.05) and marijuana (−0.22, *p* < 0.05) use. Finally, differences between the polyvictims of four types of abuse versus those who suffered five or more types of abuse were observed for the three variables analysed-alcohol (−0.25, *p* < 0.05), marijuana (−0.23, *p* < 0.05), and acceptance of violence (−0.32, *p* < 0.05).

Finally, the results of the three-step hierarchical regression model (Table 6) showed, in the first step (Model 1), that the frequency of violence had a statistically significant influence on polyvictimization (β = 0.39; *p* < 0.01). Similarly, the consumption of alcohol had a significant influence on polyvictimization (β = 0.27; *p* < 0.01). This first model obtained an R² of 0.16.

In the second step of the analysis (Model 2) (Table 6), it was found that the frequency of violence had a significant influence on polyvictimization (β = 0.41, *p* < 0.01). Similarly, the consumption of alcohol also had a significant influence on polyvictimization (β = 0.29; *p* < 0.01). Finally, the use of marijuana significantly predicted polyvictimization (β = 0.22; *p* < 0.05). The inclusion of marijuana use in this second model improved the model’s prediction, obtaining an R² of 0.24.

Finally, the third step of the hierarchical regression analysis (Model 3) (Table 6) revealed that polyvictimization was significantly predicted by the frequency of violence (β = 0.33; *p* < 0.01), the consumption of alcohol (β = 0.24; *p* < 0.05), marijuana use (β = 0.21; *p* < 0.05), and the acceptance of violence (β = 0.29; *p* < 0.01). The inclusion of the variable acceptance of violence improved the model’s variance explained, obtaining an R² of 0.31.

## 4. Discussion

The results revealed the relationships between the consumption of substances such as alcohol or marijuana, victimization in adolescent dating, and the acceptance of abuse. Likewise, the data analysis revealed that this relationship was affected by the severity (frequency) of the abuses suffered. In this sense, it was found that the adolescents who have been victimized in more than one type of abuse (polyvictims) presented different relationships with substance use and the acceptance of the said abuses depending on whether the polyvictimization occurred in two, three, four, or five or more forms of abuse.

With regard to the analysis of victimization, the data revealed that a large proportion of the adolescents who suffered some type of abuse experienced more than one type of abuse, thus becoming polyvictims. Furthermore, the abuses tended to occur at the same time, with some adolescents being polyvictims in five or more different forms of abuse. Such polyvictims could also be found within in both the group of adolescents who experienced violence “sometimes” and that of those who experienced it “frequently”. Consequently, becoming a polyvictim was not directly related to the frequency of the aggressions, but instead to the situation of being involved in an abusive relationship. This finding refers to the teen dating violence concept itself, which includes among its features the abuses experienced in different modalities (physical, psychological, emotional, etc.) [39]. When these modalities were perpetrated at the same time, the victim became a polyvictim, regardless of the frequency with which these abuses were committed.

Finding polyvictims in the study of aggression and victimization is not an isolated event. Thus, studies carried out in different countries such as the United States, Spain, or Norway have shown the presence of polyvictims in different contexts [6,40,41,42]. However, polyvictimization could be particularly relevant regarding adolescent couples whose characteristics complicate both the detection of, and intervention with, victims due to the different interpretations of abuse. Thus, while physical violence is clearly accepted by all people as being a form of severe abuse, many of the behaviours that do not involve direct physical violence or harassment may be approached with different criteria, thereby blurring the limits of what different people consider to be an abusive relationship [43]. Consequently, the greater presence of psychological or emotional violence in adolescent dating relationships [44] would contribute not only to hiding victimization but also to acceptance by the victims of this type of abuse.

From this perspective, the multiple psychological abuses that many of the polyvictims suffer (harassment, control, blackmail, emotional punishment, etc.) could at times be accepted due to the victim’s interpretation of such behaviour as being normal forms of interaction between the couple’s members. In this regard, the use of moral disengagement is key [45]. Thus, the victims seem to use such mechanisms not only to minimize the importance of the abuse, but also to justify the absence of actions aimed at resolving the problems that arise from their violent dating relationship [5], and possibly from their polyvictimization. Similarly, the normalization of violence, affected by the sexism prevailing in society, influences people by making abusive behaviour be seen as less violent or less serious if these situations are consistent with the behaviour that is expected or permitted [3].

The danger that this fact entails is reflected by the serious consequences that the phenomenon of polyvictimization has for adolescents, including the presence of both internalizing [6,46,47] and externalizing symptoms [48].

With respect to the externalizing signs, the present results indicate that the use of substances predicts polyvictimization. Thus, the results support previous studies which also pointed to this relationship when considering the influence of alcohol on the one hand [49,50] and of marijuana on the other [51,52,53,54]. With the foregoing, the joint use of the two types of substances might increase both polyvictimization and the consequent violence experienced.

The explanation for this link may be found in two different considerations. From one point of view, the use of substances by the victims could be related to coping with problems deriving from their own victimization [55,56]. Substance consumption would then be rather a consequence than a precipitating factor. This fact seems to be especially relevant in victimized adolescent girls whose risk of consuming large amounts of alcohol could be higher [55], and even be more prone to developing problems later that are related to this consumption [57]. The different habits of consumption found in the victims and aggressors seem to be more related to the two groups’ different motivations for consumption than to their role of victim or aggressor in itself [55,58].

From another point of view, the use of substances has been linked to the effects that this consumption has on people, triggering victimization and aggression. Thus, marijuana, for example, has been associated with negative effects such as increased heart rate [59], behavioural disinhibition [60], and difficulties in perception, information processing, and memory [59,61,62,63]. For its part, alcohol has been noted as having similar effects among its users to those indicated for marijuana [12,13,14]. These effects, when interacting with a context of disagreement or criticism within the couple, could foster violent behaviour, as well as a decrease in the capacity to attempt to resolve the conflict [64]. These facts could be even more serious if the couple in question has a prior history of violence and aggression [65].

Based on the above, although these two ways of explaining substance use with respect to victimization (as a predictor or consequence of violence in dating relationships) appear to be contrary in principle, they might be complementary, i.e., the consumption of alcohol and marijuana would be involved both in the onset of abuse, increasing the likelihood and severity of violent incidents [66], and in maintaining it, with the two substances being used as coping tools [55,56].

The ultimate increase in the victims’ acceptance and normalization of abuse has obvious implications for its detection and the consequent interventions. Today’s society, far from presenting an egalitarian orientation, introduces a new concept of neoliberal and globalized masculinity. This carries with it a more twisted and hidden discourse that implies a form of male control which is harder to detect [67]. Similarly, the apparent empowerment of women puts them in a misleadingly favourable liberal position in which adolescent girls, in particular, see themselves in the same position as their male peers [68], having the same kinds of roles and behaviours. However, the difference between the sexes is still present in society to the point of classifying women who consume alcohol as being a lesser degree of victim than those who do not, precisely because such consumption does not conform to the normed vision of what women’s behaviour should be [69,70].

Consequently, the continuous feedback between substance use and violence, and its acceptance, may cause some polyvictims to minimize the importance of their abuse by considering that their behaviour does not conform to what is expected of them. Indeed, it has even been noted that women who use substances and who are victims of aggression tend to be socially blamed for this abuse to a greater extent than those who do not consume that same substance, while at the same time excusing the aggressors who are considered to be less responsible [71]. These facts stand out even more when one additionally takes into account the cultural and social context. It has been pointed out how the prevailing culture in society determines the normalization of abuse to the point of finding a greater presence of interpersonal violence in societies with a strong patriarchal ideology [72,73,74]. This fact is in line with the greater presence of benevolent sexism, in which adolescent boys and girls are today both scoring increasingly higher [75]. Thus, if the acceptance of violence alone determines victimization, the joint relationship of this variable with substance use and the frequency of violence would play a key role in the establishment and maintenance of polyvictimization.

## 5. Conclusions

Adolescent dating violence is an important phenomenon that has been detected all over the world. The main objective of researchers has been to uncover the variables associated with the reasons that lead the aggressor to harass or abuse their romantic partner. The major contribution of the present study is to offer an alternative perspective focused on the victim, in particular analysing the influence that variables frequently associated with the aggressors, such as drug abuse or normalization of violence, have in the victims. Additionally, it was found that the role of victim in abusive relationships of this type often seems to be associated with polyvictimization, and that this phenomenon is precipitated by substance use, the frequency of abuse, and the acceptance of violence, in a cycle of mutual interaction. The simultaneous consideration of the relationships linking all the variables involved in adolescent dating violence not only explains the phenomenon itself but has further obvious implications for front-line professionals who are developing prevention activities and intervention programs in this phenomenon of adolescent dating violence.

## 6. Limitations

The study has some limitations. One, it was cross-sectional so that the results must be taken with caution. Two, the age of the participants was not considered as a variable. Given that they were in adolescence, the developmental moment they were living could well affect the results. Three, such constructs and variables as moral disengagement, sexism, negative emotions (low self-esteem, anxiety, insecurity, etc.), and social and cultural differences among the participants could have impacted the web of interactions created among the variables that were studied. The perspective of the aggressors and of the víctim-aggressors could also be an invaluable aspect to add into the network of variables to analyse. Likewise, it would be interesting to study whether these adolescent victims had also been victims of violence during childhood.

These limitations should orient future, deeper investigations aimed at gaining further knowledge about the phenomenon of teen dating violence, victimization, and aggression.

## Figures and Tables

**Table 1:** Modalities of victimization.

Modalities of Aggression	Victims
Detachment	60.6%
Humiliation	1.8%
Sexual abuse	19.8%
Coercion	37.2%
Gender violence	22.4%
Physical abuse	8.8%
Emotional punishment	47.3%
Instrumental punishment	9.8%

**Table 2:** Consumption of alcohol and marijuana.

	Non-victim		Victim	
	Male	Female	Male	Female
Drink	342 (66%)	413 (67.3%)	537 (85.4%)	712 (88.9%)
Do not drink	176 (34%)	201 (32.7%)	92 (14.6%)	89 (11.1%)
Total	518 (100%)	614 (100%)	629 (100%)	801 (100%)
Smoke	83 (16.2%)	80 (13.2%)	182 (29%)	239 (29.8%)
Do not smoke	429 (83.8%)	528 (86.8%)	445 (71%)	563 (70.2%)
Total	512 (100%)	608 (100%)	627 (100%)	802 (100%)

**Table 3:** Identification of polyvictims.

Number of Aggressions	Polyvictims	
	Sometimes	Frequently
Two abuses	211 (27.1%)	9 (2.2%)
Three abuses	194 (24.9%)	35 (8.6%)
Four abuses	176 (22.6%)	44 (10.8%)
Five or more abuses	197 (25.3%)	319 (78.3%)

**Table 4:** Correlation analysis.

	1	2	3	4
1. Victimization				
2. Alcohol	0.36 ***			
3. Marijuana	0.29 **	0.22 *		
4. acceptance	0.32 ***	0.20 *	0.18 *	

* *p* < 0.05, ** *p* < 0.01, *** *p* < 0.001.

**Table 5:** ANOVA and post hoc test for factor and victimization group.

	F	P	ηp^2^	Power	Post Hoc			
					V vs. Pv2	Pv2 vs. Pv3	Pv3 vs. Pv4	Pv4 vs. Pv5+
Alcohol	7.367	0.000	0.007	0.967	−0.08	−0.19 *	−0.20 *	−0.25 *
Marijuana	5.307	0.000	0.010	0.913	−0.010	−0.17	−0.22 *	−0.23 *
acceptance	2.612	0.000	0.016	0.924	−0.07	−0.21 *	−0.26 *	−0.32 *

df: 2.059; * *p* < 0.05. V: victims; Pv2: polyvictims with 2 abuses; Pv3: polyvictims with 3 abuses; Pv4: polyvictims with 4 abuses; Pv5+: polyvictims with 5 or more abuses.

**Table 6:** Hierarchical regression.

Variables	Polyvictimization					
	Model 1		Model 2		Model 3	
	β	(t)	β	(t)	Β	(t)
Frequency	0.39	(10.84 **)	0.41	(11.13 **)	0.33	(9.51 **)
Alcohol	0.27	(7.03 **)	0.29	(7.40 **)	0.24	(6.26 *)
Marijuana			0.22	(5.16 *)	0.21	(5.05 *)
acceptance					0.29	(7.54 **)
R^2^	0.16		0.24		0.31	
^⬉^R^2^			0.15		0.20	

* *p* < 0.05, ** *p* < 0.01.
